# Exploring Bioactive Metabolites From *Fusarium falciforme* and *Aspergillus terreus* Isolated From Protease‐Rich Fruits: Antifungal, Antitrypanosomal, and Enzymatic Inhibitory Activities

**DOI:** 10.1002/cbdv.202500673

**Published:** 2025-07-20

**Authors:** Gabriela de Oliveira Almeida, Vitor de Souza Mazucato, Ludmilla Tonani, Marcia Regina von Zeska Kress, Gisele Barbosa, Renata Krogh, Adriano Defini Andricopulo, Leonardo Luiz Gomes Ferreira, Paulo Cezar Vieira

**Affiliations:** ^1^ Department of BioMolecular Sciences, School of Pharmaceutical Sciences of Ribeirão Preto University of São Paulo Ribeirão Preto São Paulo Brazil; ^2^ Department of Clinical Analysis, Toxicology, and Food Science, School of Pharmaceutical Sciences of Ribeirão Preto University of São Paulo Ribeirão Preto São Paulo Brazil; ^3^ São Carlos Institute of Physics University of São Paulo São Carlos São Paulo Brazil

**Keywords:** antifungal | antiparasitic activity | phytopathogenic fungi | protease inhibition | secondary metabolites

## Abstract

Fungal secondary metabolites display remarkable chemical diversity and biological potential, with applications in agriculture and pharmaceuticals. This study isolated and characterized bioactive metabolites from two phytopathogenic fungi, *Fusarium falciforme* (from papaya) and *Aspergillus terreus* (from pineapple), assessing their antifungal and cysteine protease‐inhibitory activities. The compounds hymeglusin, fusaridioic acid A, and butyrolactone I exhibited significant antifungal effects, with hymeglusin (IC_50_ 22.12 µg/mL) inhibiting *A. terreus* and butyrolactone I (IC_50_ 39.72 µg/mL) inhibiting *F. falciforme*. These metabolites also strongly inhibited papain, with IC_50_ values of 6.24 µM (butyrolactone I), 9.38 µM (hymeglusin), and 28.58 µM (fusaridioic acid A). Butyrolactone I exhibited the highest antitrypanosomal activity (IC_50_ 24.72 µM), highlighting its antiparasitic potential. These findings emphasize the biotechnological significance of fungal metabolites for antifungal, protease‐inhibitory, and antiparasitic applications while also shedding light on their ecological role in host–pathogen interactions.

## Graphical abstract

Metabolites from fungi isolated from protease rich‐fruits displayed antifungal, antitrypanosomal and enzymatic inhibitory activities.

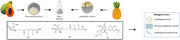

**Abbreviations**
caM: calmodulin
CDK: cyclin‐dependent kinase
CPRG: chlorophenol red‐β‐d‐galactopyranoside
DNA: deoxyribonucleic acid
E‐64: 1‐[L‐*N*‐(*trans*‐epoxysuccinyl)leucyl] amino‐4‐guanidinobutane
EF‐1α: translation elongation factor region
ESI: electrospray Ionization
FCFRP: Faculdade de Ciências Farmacêuticas de Ribeirão Preto (School of Pharmaceutical Sciences of Ribeirão Preto)
FCS: fetal calf serum
HFF‐1: human foreskin fibroblasts
HMGCS1: hydroxymethylglutaryl‐CoA synthase
HPLC‐DAD: high‐performance liquid chromatography with diode array detection
HRMS: high‐resolution mass spectrometry
IC_50_
: 50% inhibitory concentration
LMC: Laboratório de Micologia Clínica
MCA: 7‐amino‐4‐methylcoumarin
NCBI: National Center for Biotechnology Information
NMR: nuclear magnetic resonance
PCR: polymerase chain reaction
PDA: potato dextrose agar
RP: reverse phase
Rt: retention time
RPB2: DNA‐dependent RNA polymerase II
TLC: thin‐layer chromatography
TOF: time‐of‐flight
USP: University of São Paulo
VLC: vacuum liquid chromatography

## 1 Introduction

Special metabolites produced by fungi exhibit high chemical and biological diversity, reflecting their potential for addressing various human challenges, ranging from the biological control of agricultural pests to the development of drugs for numerous diseases [1, 2].

Agriculture is the basis of many countries’ economies and the foundation of human sustenance and survival worldwide [3]. Thus, pest control is essential to reduce production losses, which has led to the development and use of chemical fertilizers and pesticides for many years. However, the widespread use of these chemicals has had harmful effects, including the development of resistance, soil and water contamination, and risks to human health [4]. Considering these challenges and the increased agricultural demand due to global population growth, the search for biological agents for pest control has intensified. Biopesticides are considered an environment‐friendly management strategy, as they are less toxic to living organisms, exhibit diverse modes of action, are more selective for pathogens, and are naturally biodegradable. In this context, fungal secondary metabolites have gained prominence in the production of microbial pesticides for controlling diseases in food crops [5].

Beyond their agricultural applications, fungal metabolites are being extensively explored for the development of pharmaceuticals to treat various pathologies, including antimicrobials, neuroprotective agents, antiparasitic drugs, antioxidants, antitumor agents, and anti‐inflammatory compounds [6]. An area that remains underexplored is the study of fungal secondary metabolites that inhibit cysteine proteases. Cysteine proteases participate in various biological processes, and their imbalance is associated with increased proteolytic activity, which leads to diseases such as osteoporosis, arthritis, Alzheimer's disease, and cancer [7, 8, 9]. Furthermore, certain cysteine proteases are critical for the life cycle and pathogenicity of some parasites, such as *Trypanosoma cruzi* (cruzain) [10] and *Plasmodium* spp. (falcipains 2 and 3) [11], playing roles in immune evasion, enzymatic activation, virulence, tissue and cell invasion, excystation, hatching, and molting [12]. Similarly, cysteine proteases have been implicated in the virulence of viruses such as SARS‐CoV‐2 (PL1pro, PL2pro, and Mpro) [13], Chikungunya, and Mayaro viruses (nsP2) [14], participating in processes such as host cell entry and viral genome replication. Therefore, the enzymatic inhibition of proteases is a promising strategy for developing new drugs.

Fruits such as papaya (*Carica papaya*) and pineapple (*Ananas comosus*) have high protease content [15], including papain, chymopapain, caricain, and bromelain. In fruits and plants, these enzymes play a crucial role in defense against pathogens, including fungi, larvae, and other pests [16, 17, 18]. However, some pathogenic fungi can infect these fruits, possibly indicating their ability to inhibit these proteases. *Fusarium proliferatum* is known for synthesizing secondary metabolites, such as the mycotoxins fusaric acid and beauvericin, which inhibit papain and may play a role in its infection mechanism in pineapple [19]. Other cysteine protease inhibitors have been isolated from microorganisms, the most notable being E‐64 (1‐[l‐*N*‐(*trans*‐epoxysuccinyl)leucyl] amino‐4‐guanidinobutane), an irreversible inhibitor isolated from the fungus *Aspergillus japonicus* [20].

In this context, the present study aimed to isolate and characterize metabolites produced by phytopathogenic fungi isolated from papaya (*Fusarium falciforme*) and pineapple (*Aspergillus terreus*), with antifungal and cysteine protease‐inhibitory potential for future biotechnological applications. Additionally, this study explores whether fungi inhabiting protease‐rich environments produce metabolites capable of inhibiting cysteine proteases, providing valuable insights into the chemical strategies developed by these microorganisms and their potential translational applications.

## 2 Results and Discussion

### 2.1 Identification of Isolated Fungal Species

Fungi are well recognized for their ability to produce specialized metabolites with diverse biotechnological applications in the pharmaceutical, agricultural, food, and cosmetic industries [6]. In this study, we isolated fungal species from decayed papaya and pineapple fruits to identify bioactive compounds with therapeutic potential. Multiple fungal isolates were obtained and identified using both classical and molecular methods. Among them, due to their promising metabolic profiles, our focus was directed toward one phytopathogenic fungus isolated from papaya and another phytopathogenic fungus from pineapple.

**FIGURE 1:**
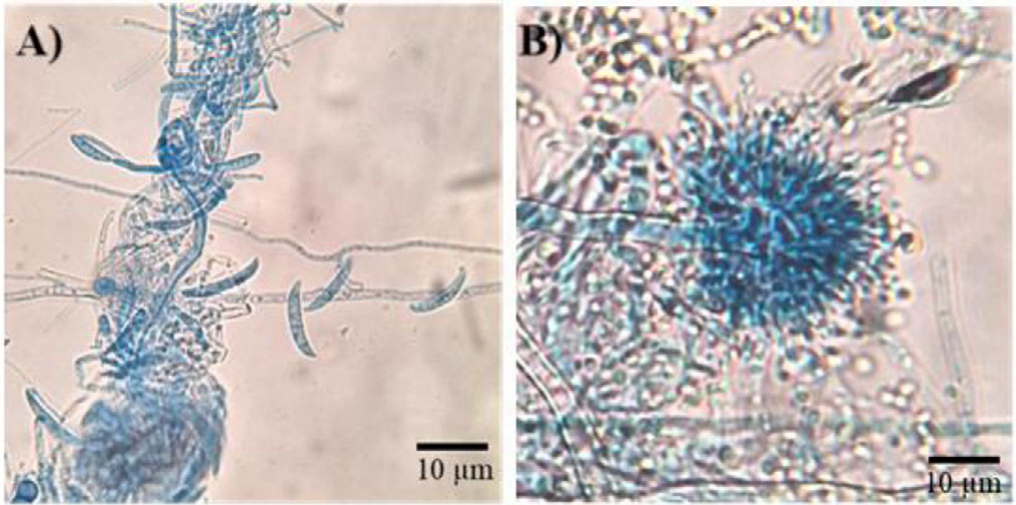
Micromorphology of fungi isolated from (A) papaya (*Fusarium falciforme*) and (B) pineapple (*Aspergillus terreus*). Samples were stained with lactophenol cotton blue and observed under bright‐field microscopy at 400× magnification. Scale bar: 10 µm.

**FIGURE 2:**
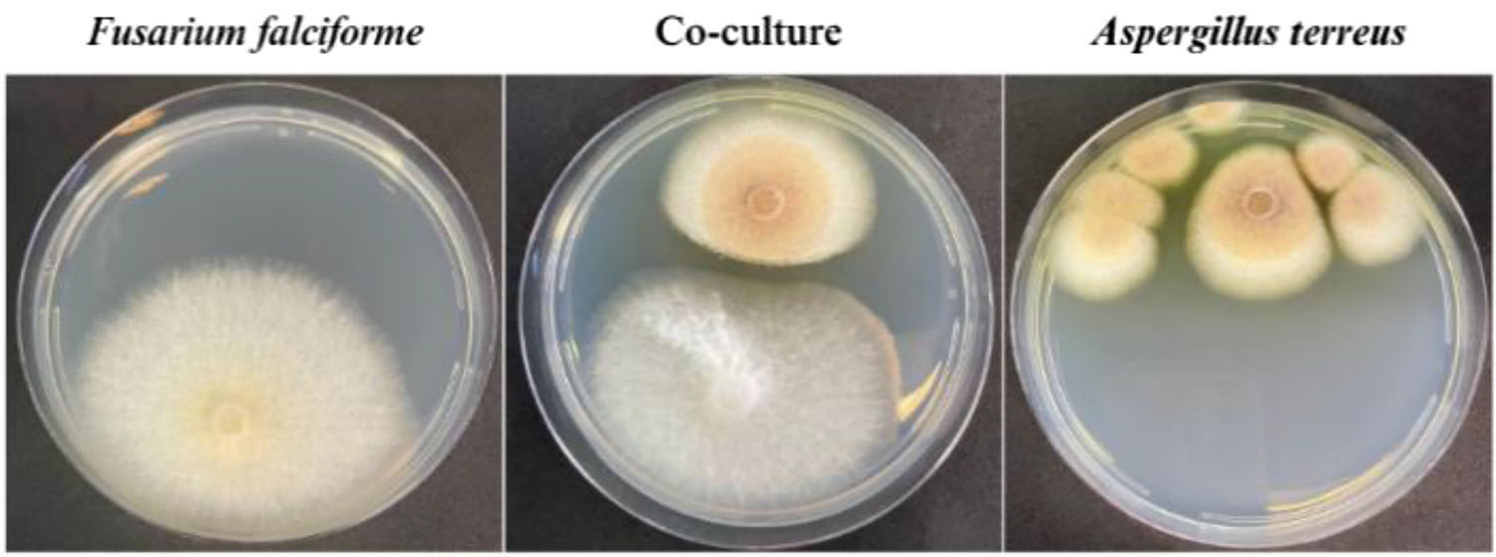
Axenic and coculture growth of *Fusarium falciforme* LMC23007.2 and *Aspergillus terreus* LMC23020 on PDA medium incubated at 27°C for 7 days. The left panel shows *F. falciforme* under axenic conditions, exhibiting uniform radial growth with a well‐defined margin and a slightly yellowish center. The right panel displays *A. terreus* in axenic culture, characterized by multiple colonies with distinct yellowish to brownish pigmentation and a textured surface. The central panel illustrates the coculture condition, where *A. terreus* exhibits an antagonistic interaction against *F. falciforme*, forming a well‐defined inhibition zone with reduced mycelial density at the interaction interface.

The isolated fungi were cultured on potato dextrose agar (PDA) medium for micromorphological (Figure 1) and macromorphological characterization (Figure 2). The fungus isolated from rotten papaya exhibited aerial mycelium ranging from white to cream in color (Figure 2). Its conidiophores produced hyaline, two‐celled, fusiform macroconidia, along with rare rough‐walled chlamydospores (Figure 1A). These morphological features are characteristic of species within the *Fusarium* genus [21]. The fungus isolated from rotten pineapple formed colonies with a suede‐like texture, displaying a light yellow to cinnamon‐buff coloration that gradually darkened to brown (Figure 2). The hyaline conidiophore featured a compact, biseriate globose conidiogenous head, from which globose‐shaped conidia emerged, aligning with morphological traits characteristic of the *Aspergillus* genus (Figure 1B) [22].

Molecular identification, achieved through sequencing of the target region and genes, confirmed the species previously determined based on the isolates’ morphological characteristics. *F. falciforme* LMC23007.2 was isolated from papaya, and *A. terreus* LMC23020 from pineapple (Table S1).

The genus *Fusarium* has been extensively explored due to its potential to yield bioactive compounds with potential applications in treating various diseases [23], including microbial infections [24, 25], parasitic diseases [26, 27], autoimmune disorders [28, 29], and cancer [30]. *F. falciforme* is a member of the *Fusarium solani* species complex (FSSC) [31]. Although the metabolic profile of the *Fusarium* genus is well documented, relatively few studies have specifically examined the metabolites produced by *F. falciforme*. Conversely, *A. terreus* is a globally distributed fungus and a significant species within the *Aspergillus* genus, renowned for its ability to produce a wide array of secondary metabolites, which supports its extensive biotechnological applications. However, it is also associated with considerable environmental, ecological, economic, and human health impacts [32, 33].

To assess the impact of *F. falciforme* LMC23007.2 on the growth of *A. terreus* LMC23020, both fungal isolates were cultured on PDA medium in petri dishes under axenic and coculture conditions. The fungal colonies appeared to have a well‐defined margin and homogenous radial expansion in the axenic cultivations, suggesting a consistent growth rate without competition. Under coculture conditions, a clear antagonistic interaction between the two fungal isolates was observed, with one colony exhibiting growth inhibition in proximity to the other. The upper colony displayed a well‐defined inhibition zone, characterized by a clear boundary and reduced mycelial density at the interaction interface. This suggests the production of diffusible antifungal metabolites or competition for nutrients. The lower colony, in contrast, shows restricted growth near the upper colony, further supporting the presence of antagonistic effects (Figure 2), which suggests the production of metabolites with antifungal activity by one of the present fungi [34].

### 2.2 Analysis of Ethyl Acetate Extract

The fungi were cultivated in six Erlenmeyer flasks containing rice medium for 21 days (Section 4.3) under both axenic and coculture conditions. At the end of the fermentation period, fungal metabolites were extracted with ethyl acetate (Section 4.4). The resulting extracts, designated as R20 (*A. terreus*), R7B (*F. falciforme*), and R7B,20 (coculture of *A. terreus* and *F. falciforme*), underwent vacuum liquid chromatography (VLC) with a solvent gradient of 95:5 hexane:ethyl acetate to remove triglycerides from the rice medium and were analyzed by ^1^H NMR. The chemical profiles of the extracts are compared in Figure S2. In addition, the extracts were analyzed by high‐resolution LC–MS to complement the NMR data (Figure S3).

The spectra obtained (Figure S2) indicate that the chemical profile of the coculture extract (R7B,20) closely resembles that of the axenic culture extract of *A. terreus* (R20). This observation is corroborated by the LC–MS profiles (Figure S3), which further demonstrate that most of the major metabolites present in the coculture are shared with the axenic culture of *A. terreus*, with only a few features related to *F. falciforme* being detected. These results suggest that the metabolism of *A. terreus* dominates that of *F. falciforme* during simultaneous cultivation.

Although *A. terreus* is often reported to dominate cocultures due to its fast growth and secretion of bioactive metabolites, such dominance is not universal. For instance, in cocultures with *Streptomyces noursei*, *A. terreus* outcompetes the actinomycete; however, when paired with *Streptomyces rimosus*, it is the actinomycete that prevails [35, 36]. Additionally, antagonistic potential can vary even within the *Aspergillus* genus. In a study evaluating inhibition of *Macrophomina phaseolina*, *Agyneta flavipes* showed the highest suppression (53%), whereas *A. terreus exhibited one of the lowest levels*, reducing fungal growth by only 5%, slightly above *Aspergillus sydowii* (6%) [37]. These findings highlight that the competitive behavior of *A. terreus* is context‐dependent and influenced by both the cocultured species and intra‐generic variability within *Aspergillus*.

### 2.3 Isolation and Structural Identification of Compounds

**FIGURE 3:**
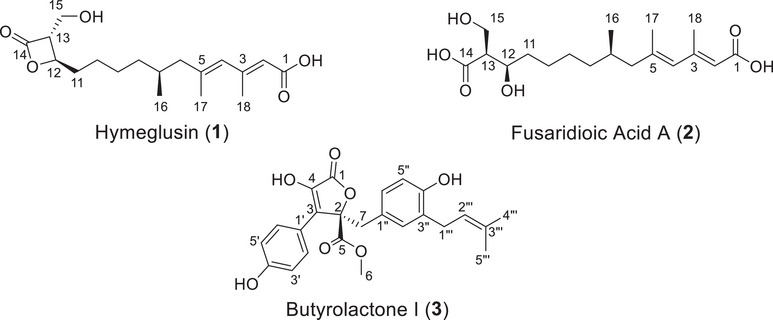
Chemical structures of compounds **1–3**.

The extracts were fractionated as detailed in Section 4.4. Each solvent fraction was concentrated under reduced pressure and evaluated for antifungal activity. Fractions from the R7B extract were tested against *A. terreus*, whereas fractions from the R20 extract were tested against *F. falciforme*. Fraction 3 from R7B (f3R7B) demonstrated the highest inhibitory activity, achieving 94.97% ± 3.31% (0.25 mg/mL), followed by fraction 4 (f4R7B) with 80.40% ± 2.21%. From the R20 extract, fraction 3 (f3R20) exhibited the highest activity, with 58.23% ± 3.16% (0.25 mg/mL) inhibition against the tested strain. The most active fractions were subjected to purification, yielding 33.3 mg of hymeglusin (**1**), 27.7 mg of fusaridioic acid A (**2**), and 94.8 mg of butyrolactone I (**3**). The pure compounds were structurally identified and further analyzed for their biological activities. The ^1^H NMR data are presented in Figures S4, S6, and S8, and the HRESIMS spectrum in Figures S5, S7, and S9. The ^1^H NMR spectroscopic data are summarized in Table S2, and the structures of the compounds are shown in Figure 3.

Hymeglusin (**1**): 33.3 mg, Rt 13.9 min. UV (MeOH) *λ*max 271 nm. ^1^H NMR (500 MHz, methanol‐d4) *δ*H 5.77 (br.s, 1H), 5.64 (br.s, 1H), 4.56 (ddd, *J* = 7.4, 6.1, 4.1 Hz, 1H), 3.88 (dd, *J* = 11.9, 4.7 Hz, 1H), 3.77 (dd, *J* = 11.9, 3.6 Hz, 1H), 3.46 (ddd, *J* = 4.7, 4.1, 3.6 Hz, 1H), 2.20, d (1.1 Hz, 3H), 2.14 (dd, *J* = 13.1, 6.5 Hz, 1H), 1.90 (m, 1H), 1.87 (m, 1H), 1.82, d (1.1 Hz, 3H), 1.77 (m, 1H), 1.72 (dd, *J* = 13.1, 6.5 Hz, 1H), 1.44 (m, 2H), 1.42 (m, 1H), 1.39 (m, 1H), 1.36 (m, 1H), 1.18 (m, 1H), 0.88 (d, *J* = 6.6 Hz, 3H). HRMS (ESI): *m/z* calcd for C_18_H_28_O_5_H^+^: 325.2015 [M + H]^+^; found: 325.2001 (error −4.3050 ppm).

Fusaridioic acid A (**2**): 27.7 mg, Rt 11.9 min. UV (MeOH) *λ*max 259 nm. ^1^H NMR (500 MHz, methanol‐d4) *δ*H 5.76 (br.s, 1H), 5.64 (br.s, 1H), 3.88 (m, 1H), 3.82 (dd, *J* = 10.6, 8.0 Hz, 1H), 3.75 (dd, *J* = 10.6, 5.4 Hz, 1H), 2.61 (ddd, *J* = 11.3, 8.0, 5.4 Hz, 1H), 2.20 (d, *J* = 1.1 Hz, 3H), 2.13 (dd, *J* = 13.3, 5.9 Hz, 1H), 1.88 (dd, *J* = 13.3, 8.4 Hz, 1H), 1.81 (d, *J* = 1.1 Hz, 3H), 1.71 (m, 1H), 1.54 (m, 1H), 1.49 (m, 1H), 1.47 (m, 1H), 1.38 (m, 3H), 1.34 (m, 1H), 1.15 (m, 1H), 0.86 (d, *J* = 6.6 Hz, 3H). HRMS (ESI): *m/z* calcd for C_18_H_30_O_6_Na^+^: 365.1940 [M + Na]^+^; found: 365.1954 (error 3.8336 ppm).

Butyrolactone I (**3**): 94.8 mg, Rt 9.4 min. UV (MeOH) λmax 227 and 306 nm. ^1^H NMR (500 MHz, methanol‐d4) δH 7.59 (d, *J* = 8.9 Hz, 2H), 6.87 (d, *J* = 8.9 Hz, 2H), 6.54 (dd, *J* = 8.2, 2.0 Hz, 1H), 6.49 (d, *J* = 8.2 Hz, 1H), 6.40 (d, *J* = 2.0 Hz, 1H), 5.06 (m, 1H), 3.78 (s, 3H), 3.46 (q, *J* = 14.7 Hz, 1H), 3.41 (d, *J* = 14.7 Hz, 1H), 3.07 (m, 2H), 1.67 (s, 3H), 1.57 (s, 3H). HRMS (ESI): *m/z* calcd for C_24_H_24_O_7_H^+^: 425.1600 [M + H]^+^; found: 425.1595 (error −1.176028 ppm).

In this study, hymeglusin and fusaridioic acid A were isolated from the cultivation of *F. falciforme* in rice, whereas butyrolactone I was obtained from the cultivation of *A. terreus* in rice. Hymeglusin is a long‐chain fatty acid first described in 1970 as Antibiotic 1233A [38]. As a specific inhibitor of hydroxymethylglutaryl‐CoA synthase (HMGCS1), it blocks the mevalonate pathway and is known for its ability to inhibit cholesterol biosynthesis. This property is linked to its adjuvant role in the therapy of certain cancers, such as acute myeloid leukemia [39, 40]. Fusaridioic acid A is an alkenoic acid first isolated in 2018 from *F. solani* [41]. It shares a long‐chain fatty acid structure similar to hymeglusin but features an open β‐lactone ring. Although little is known about its biological activity, it has been noted that the β‐lactone ring with the hydroxymethyl group in hymeglusin is essential for potent inhibition of HMG‐CoA synthase [42]. Butyrolactone I, a selective inhibitor of the cyclin‐dependent kinase (CDK) family, is one of the major secondary metabolites produced by *A. terreus*. It was first described in 1977 [43]. This compound exhibits diverse bioactivities, including antitumor [44, 45], antiviral [46], antimicrobial [47], antioxidant, and antidiabetic properties [48]. However, no reports were found regarding its activity against *F. falciforme*.

### 2.4 Antifungal Activity of Pure Compounds

**FIGURE 4:**
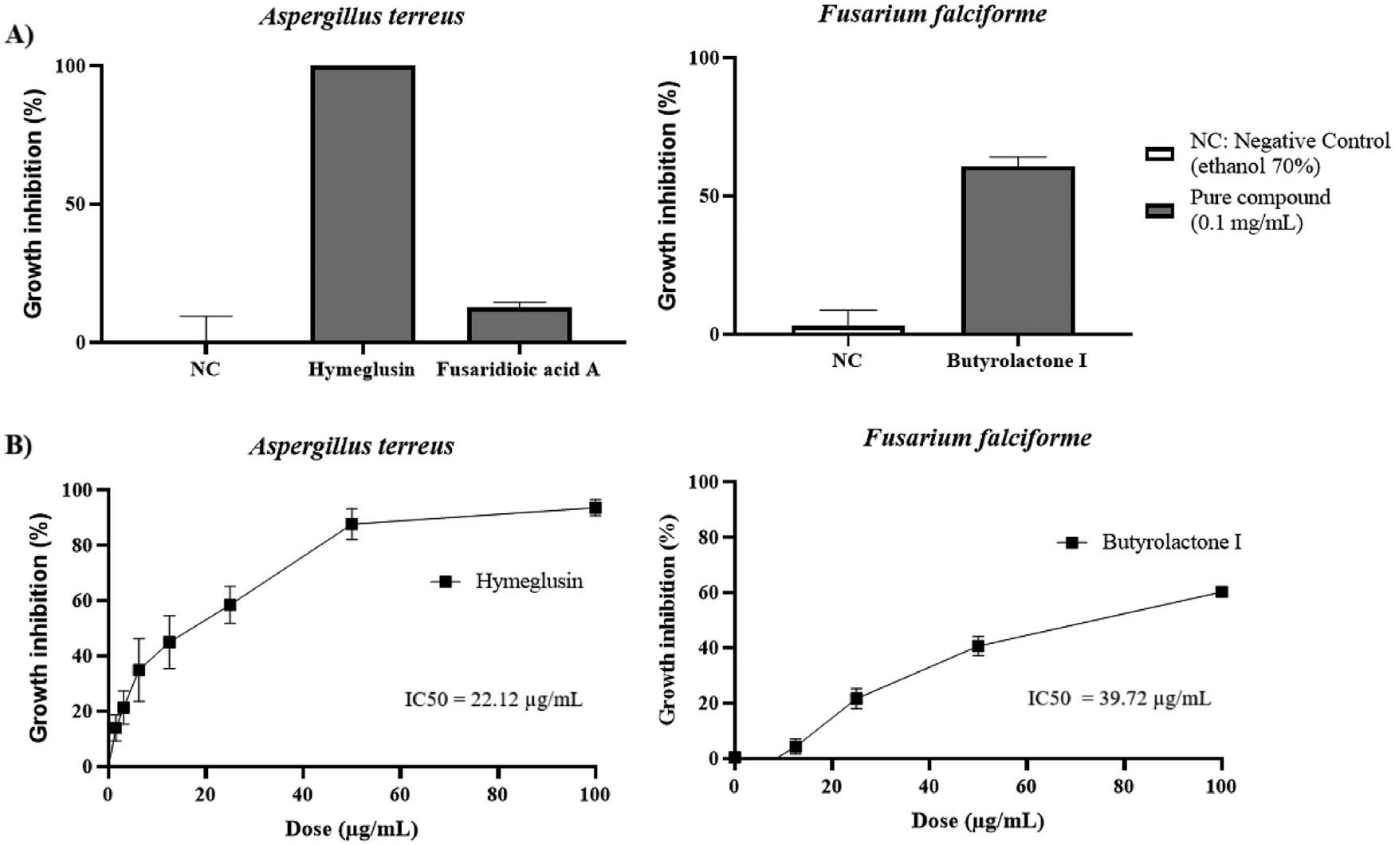
Antifungal activity of isolated compounds. (A) Growth inhibition activities in *Aspergillus terreus* by compound **1** (hymeglusin, 0.1 mg/mL) and compound **2** (fusaridioic acid A, 0.1 mg/mL), and in *Fusarium falciforme* by compound **3** (butyrolactone I, 0.1 mg/mL). Growth inhibition is expressed as a percentage relative to the untreated control (NC, negative control with solvent 70% ethanol). (B) Dose‐dependent curves used to determine IC_50_ values for hymeglusin against *A. terreus* and butyrolactone I against *F. falciforme*. The dose–response curves were fitted using a nonlinear regression model (log[inhibitor] vs. normalized response) to determine the concentration of each compound required to inhibit fungal growth by 50%. Data represent the mean ± standard deviation (SD) from two independent experiments conducted in triplicate.

The antifungal activity of compounds **1** and **2**, isolated from *F. falciforme*, against *A. terreus*, and compound **3**, isolated from *A. terreus*, against *F. falciforme*, was evaluated (Section 4.6). The results (Figure 4A) demonstrate that hymeglusin completely inhibited the growth of *A. terreus* (100% ± 0.00%) at a concentration of 0.1 mg/mL, whereas fusaridioic acid A exhibited only 12.71% ± 1.75% inhibition. This significant reduction in antifungal activity highlights the critical role of the lactone ring in the compound's bioactivity, as its hydrolysis markedly diminished its efficacy. Butyrolactone I displayed notable antifungal activity against *F. falciforme*, achieving 60.70% ± 3.44% growth inhibition at 0.1 mg/mL. On the basis of these results, compounds **1** and **2** were further analyzed to determine the concentration required to inhibit fungal growth by 50% (Figure 4B).

The antifungal activity of the isolated compounds revealed that hymeglusin (IC_50_ 22.12 µg/mL) and butyrolactone I (IC_50_ 39.72 µg/mL) exhibited potent inhibitory activity against *A. terreus* and *F. falciforme*, respectively. These findings highlight these substances’ promising potential for future applications as antifungal agents in human health or agriculture. Plant diseases caused by phytopathogenic fungi significantly threaten global food security by compromising agricultural productivity and food quality. The indiscriminate use of chemical fungicides has contributed to the emergence of microbial resistance while also posing risks to human health and the environment [4]. In this context, naturally derived secondary metabolites have emerged as promising resources for the development of more sustainable biopesticides. Fungi are a rich source of antifungal compounds that play a crucial role in their ecology, enabling them to survive across diverse niches [5]. These metabolites contribute to resource competition, inhibit the growth of competing microorganisms, and serve as defense mechanisms against predators [49]. This study's findings highlight the potential of hymeglusin, a secondary metabolite produced by *F. falciforme*, as an effective antifungal agent against *A. terreus*, a phytopathogen affecting pineapple crops. Similarly, butyrolactone I, produced by *A. terreus*, exhibited potent antifungal activity against *F. falciforme*, a phytopathogen responsible for diseases in papaya.

Comparable studies in the literature reinforce the relevance of our findings. For instance, javanicin, a compound isolated from *Fusarium sp*., exhibited strong activity against *Candida albicans* (IC_50_ = 6.16 µg/mL), whereas cyclic peptides such as enniatin and beauvericin showed MIC values ranging from 1.5 to 12.5 µM against *C. albicans* but lacked efficacy against *Aspergillus flavus*, *Aspergillus niger*, and *Aspergillus ochraceus* filamentous fungi [50, 51]. Additionally, 1‐methyl emodin and terrein, isolated from *A. terreus* associated with the gut of a dragonfly, demonstrated potent inhibitory activity against *Alternaria solani* and 1‐methyl emodin against *Fusarium oxysporum* f. sp. *cucumerinum*, with IC_50_ values below 0.1 µg/mL. However, these same compounds showed only moderate activity against *F. oxysporum* f. sp. *momordicae* and *Fusarium graminearum*, whereas other metabolites from the same study showed no antifungal activity at all [52]. Taken together, these comparative data illustrate the highly variable antifungal potential of fungal metabolites depending on the target organism and position hymeglusin and butyrolactone I as promising candidates within this complex landscape. Our results underscore the ecological significance of secondary metabolites in fungal interactions and their biotechnological potential for controlling fungal diseases in fruit crops, offering safer and more sustainable alternatives to conventional management practices.

### 2.5 Protease Inhibition Activity of Pure Compounds

**FIGURE 5:**
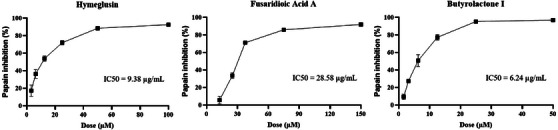
Determination of IC_50_ values for enzymatic inhibition of papain by hymeglusin, fusaridioic acid A, and butyrolactone I. The IC_50_ values were calculated using nonlinear regression analysis based on the dose–response model. Each experiment was performed in triplicate and repeated independently twice.

A fluorimetric assay was conducted to evaluate the ability of extracts and compounds to inhibit the enzymatic activity of papain, a cysteine protease (Section 4.7). This assay serves as a preliminary screening to assess these compounds’ potential for future applications in treating diseases involving cysteine proteases, such as neglected tropical diseases (NTDs) and certain viral infections. The principle of the method lies in monitoring the increase in fluorescence resulting from the cleavage of the ZFR‐MCA substrate by papain. In the presence of an inhibitor, such as E‐64 (an irreversible inhibitor used as a positive control), papain activity is suppressed. No fluorescence increase is observed, as the ZFR‐MCA substrate remains uncleaved. A fluorimetric assay was conducted with ethyl acetate extract and compounds **1–3**. The R7B extract exhibited an inhibition of 68.37% ± 0.01% at 50 µg/mL, whereas the R20 extract inhibited 102.82% ± 2.96% at the same concentration. The extract results demonstrated that both exhibit protease‐inhibitory activity, with the R20 extract showing greater potential. The isolated compounds were evaluated at initial concentrations of 50, 100, and 150 µM to determine the concentration at which papain inhibition approached 100%. The results revealed that hymeglusin achieved near‐complete inhibition at 100 µM, fusaridioic acid A at 150 µM, and butyrolactone I at 50 µM. On the basis of these findings, each compound's dilution series was performed to calculate the IC_50_ (Figure 5). For method validation, the irreversible cysteine protease inhibitor E‐64 was tested at different concentrations as described in the methodology, leading to 90% inhibition at 1 µM (25 nM in the well), thus confirming the assay's reliability.

The present study was conducted with two fungal strains isolated from fruits known for their high levels of cysteine proteases, enzymes that play a crucial role in defense against pathogens [53, 54]. Papaya latex is primarily composed of four major cysteine proteases: papain, chymopapains A and B, and papaya peptidase [55]. In pineapple, the predominant protease is bromelain, although other proteolytic enzymes, such as ananain and comosain, are present in smaller amounts [56]. Here, papain was used as a model cysteine protease to evaluate the inhibitory potential of the isolated compounds against this class of enzymes. The results demonstrated that butyrolactone I (IC_50_ 6.24 µM) and hymeglusin (IC_50_ 9.38 µM) exhibited potent inhibitory activity against papain. Fusaridioic acid A (IC_50_ 28.58 µM) also showed notable inhibitory activity, although it was less potent. The synthesis of specialized metabolites with protease‐inhibitory activity may be an adaptive strategy that enables these microorganisms to colonize and infect fruits rich in proteases, which function as a natural defense mechanism against microbial invasion [17, 18]. Thus, an effective strategy for isolating and obtaining these compounds is the cultivation of phytopathogenic fungi derived from plant hosts with high protease levels, including fruits and other protease‐rich tissues.

Despite the biological relevance of cysteine protease inhibition, studies evaluating the inhibitory activity of fungal metabolites against papain remain scarce. One of the few reports available describes the isolation of compounds from *F. proliferatum* associated with pineapple, in which beauvericin, *N*‐ethyl‐3‐phenylacetamide, and the cyclodipeptide cyclo(l‐Leu‐l‐Pro) displayed significant inhibitory effects against papain, with IC_50_ values of 25.3 ± 1.9, 39.4 ± 2.5, and 7.4 ± 0.5 µM, respectively [19]. These values are comparable to those observed in the present study reinforcing the potential of fungal metabolites as sources of cysteine protease inhibitors. Such findings underscore the promise of fruit‐associated fungi in yielding bioactive compounds capable of modulating proteolytic activity. Even so, much remains to be explored regarding the structural diversity and biological mechanisms of fungal‐derived protease inhibitors.

This study represents the first report on the inhibitory activity of hymeglusin, fusaridioic acid A, and butyrolactone I against papain and contributes new evidence to this emerging field and supports the continued investigation of fungal secondary metabolites as promising scaffolds for the development of therapeutic agents targeting cysteine proteases. The ability of these compounds to inhibit proteases suggests potential applications in the treatment of specific diseases, given that cysteine proteases play a crucial role in the virulence cycles of various pathogens, including Chikungunya and Mayaro viruses [14], as well as SARS‐CoV‐2 [13]. Additionally, cysteine proteases play a key role in the pathogenicity of protozoa, including *Plasmodium* spp. (the causative agent of malaria) [11], *Leishmania* spp. (the causative agent of leishmaniasis) [56], and *T. cruzi* (the causative agent of Chagas disease) [10]. As the isolated compounds inhibited papain activity, their antitrypanosomal potential was also evaluated.

### 2.6 Anti‐*Trypanosoma Cruzi* Activity of Pure Compounds

The assay was performed as described in Section 4.8. The IC_50_ values determined for hymeglusin (52.54 µM), fusaridioic acid A (60.07 µM), and butyrolactone I (24.72 µM) corroborate the results obtained in the enzymatic assays (Section 2.5). Among the compounds analyzed, butyrolactone I exhibited the highest inhibitory potency against papain. For comparison, benznidazole was used as a positive control and displayed an IC_50_ of 2.24 µM. The results suggest a relationship between protease inhibition by the evaluated compounds and antitrypanosomal activity. This could be explained by the presence of cruzain, a cysteine protease essential for the survival, replication, and host interaction of *T. cruzi*, in all stages of the parasite's life cycle.

Previous studies have also highlighted the potential of fungal metabolites as antiparasitic agents. Beauvericin, isolated from *Fusarium sp*., demonstrated potent activity against *T. cruzi* with an IC_50_ of 2.43 µM, a value comparable to that of the reference drug benznidazole (IC_50_ = 2.24 µM) [26]. In contrast, fumitremorgin C, a compound obtained from the methanolic extract of *Aspergillus fumigatus*, exhibited moderate trypanocidal activity (IC_50_ = 9.6 µM), whereas pseurotin D showed no significant activity (IC_50_ > 50 µM) [57]. These findings reflect the structural diversity and variable bioactivity of fungal metabolites against protozoan parasites. Although the compounds evaluated in the present study showed less potent inhibition of *T. cruzi* cysteine protease activity compared to benznidazole or beauvericin, their ability to inhibit papain and correlate with antitrypanosomal effects reinforces the hypothesis that cysteine proteases such as cruzain are plausible molecular targets. Moreover, these results underscore the relevance of exploring fungal secondary metabolites as scaffolds for the development of new therapeutics against Chagas disease.

## 3 Conclusions

In conclusion, this study demonstrates the biotechnological relevance of the metabolites hymeglusin, fusaridioic acid A, and butyrolactone I, which showed significant antifungal, cysteine protease‐inhibitory, and antitrypanosomal activities. Notably, this is the first report describing the inhibitory activity of these compounds against papain, suggesting their potential as lead molecules for the development of protease‐targeted antiparasitic or antimicrobial agents. Furthermore, these findings expand the known metabolic repertoire of *F. falciforme* and *A. terreus*, reinforcing the importance of fungal metabolites as valuable sources for pharmaceutical and agricultural applications. Future studies should focus on elucidating their mechanisms of action, exploring structure–activity relationships, and assessing their efficacy in biological models.

## 4 Experimental Section

### 4.1 Isolation of Fungal Species From Fruits With High Cysteine Protease Content

Phytopathogenic fungi were isolated from fresh fruits of papaya (*C. papaya*) and pineapple (*A. comosus*). The fruits were washed under running water to remove surface residues and subsequently stored in sterile, sealed glass containers for 21 days (papaya) and 28 days (pineapple). The incubation was performed at 27°C. At the end of the incubation period, fungal colonies present on the fruits were transferred to petri dishes containing PDA supplemented with chloramphenicol (200 mg/L). The colonies grown were isolated using the streak‐plate method. The isolated fungi were deposited in the culture collections of the Laboratório de Micologia Clínica (LMC)‐FCFRP/USP, Brazil.

### 4.2 Phenotypic and Molecular Identification of Isolated Fungi

The morphological characterization of the isolated fungi was conducted through macro‐ and micromorphological analyses. For macromorphological characterization, 2 µL of fungal conidia suspensions was inoculated at the center of sterile PDA plates and incubated at room temperature for 7 days. The colonies’ characteristics, including their appearance from both the front and reverse sides, were observed. The micromorphological analysis was performed using the microculture method. A small fragment (5 × 5 mm^2^) of PDA was placed on a sterilized microscope slide, and 2 µL of conidial fungal suspension was inoculated onto the PDA surface. A sterile coverslip was placed over the inoculum. The slide was positioned inside a sterile glass petri dish on a V‐shaped twisted glass rod, with a filter paper soaked in autoclaved water placed beneath it to create a humid chamber [58, 59, 60]. The system was incubated at 28°C until the formation of asexual reproductive structures. Afterward, the coverslip was removed and transferred to a microscopic slide containing lactophenol cotton blue solution. The fungal structures, such as conidia, conidiophores, and hyphae, were assessed under an optical microscope at 400× magnification (Observer Z1—Carl Zeiss Jena, Germany).

For molecular identification, fungal DNA was extracted using the phenol‐chloroform method as described by Tonani et al., following grinding with a pestle and mortar in liquid nitrogen [61]. The internal transcribed spacer (ITS) region, specifically ITS1‐5.8S‐ITS2 of nuclear ribosomal DNA, was amplified by polymerase chain reaction (PCR) using GoTaq Hot Start Polymerase (Promega, EUA) and the primers ITS1 (5′‐TCCGTAGGTGAACCTGCGG‐3′) and ITS4 (5′‐TCCTCCGCTTATTGATATGC‐3′) [62]. To achieve species‐level identification of *Aspergillus*, amplification of the partial sequences of calmodulin (caM) and β‐tubulin genes was performed using the primers cmd5 (5′‐CCGAGTACAAGGAGGCCTTC‐3′) and cmd6 (5′‐CCGATAGAGGTCATAACGTGG‐3′) [63], as well as bt2a (5′‐GGTAACCAAATCGGTGCTGCTTTC‐3′) and bt2b (5′‐ACCCTCAGTGTA GTGACCCTTGGC‐3′) [64, 65]. For *Fusarium* species identification, the partial sequence of the DNA‐dependent RNA polymerase II (RPB2) and translation elongation factor (EF‐1α) genes were amplified using the primers RPB2‐5f2 (5′‐GGGGWGAYCAGAAGAAGGC‐3′) [66], fRPB2‐7cR (5′‐CCCATRGCTTGYTTRCCCAT‐3′) [67], EF1‐728F (5′‐CAT CGA GAA GTT CGA GAA GG‐3′), and EF1‐986R (5′‐TAC TTG AAG GAA CCC TTA CC‐3′) [68].

PCR products were purified using the Wizard SV Gel and PCR Clean‐Up Kit (Promega, EUA). The purified DNA fragments were analyzed using 1.0% agarose gel electrophoresis and sequenced using the same primers in the ABI3730xl Genetic Analyzer (Applied Biosystems). The resulting sequences were analyzed using ChromasPro software version 2.1.10 (Technelysium Pty Ltd., Tewantin, Queensland, Australia). The obtained sequences were compared against the genetic sequence databases (GenBank) from the National Center for Biotechnology Information (NCBI) using the BLASTn approach.

### 4.3 Cultivation of Isolated Fungi Species

The fungal species isolated from papaya (*F. falciforme* LMC23007.2) and pineapple (*A. terreus* LMC23020) were inoculated onto PDA medium and incubated at 27°C for 7 days, undergoing two subcultures. Subsequently, six mycelial agar plugs (diameter 0.5 cm) were transferred into a rice medium consisting of 90 mg of parboiled rice and 90 mL of ultrapure water. Cocultivation of the two species was also performed to analyze their interaction. For large‐scale production, six Erlenmeyer flasks were prepared for each culture condition. All cultures, including monocultures and cocultures, were incubated under static conditions, in the absence of light, at 27°C for 21 days.

### 4.4 Extraction and Isolation of Special Metabolites

At the end of the fermentation period, fungal metabolites were extracted using ethyl acetate (analytical grade) as the solvent. A volume of 150 mL of ethyl acetate was added to each Erlenmeyer flask and homogenized with a glass rod to ensure thorough contact between the solvent and fungal biomass. Subsequently, the flasks were subjected to ultrasonication for 5 min to enhance extraction efficiency. The resulting extract was filtered and collected. This extraction procedure was repeated three times per flask, yielding a total volume of 450 mL of crude extract per Erlenmeyer and 2.7 L per sample, which was then concentrated under reduced pressure using a rotary evaporator. The final crude extracts obtained were designated R7B (2.58 g), R20 (8.11 g), and R7B,20 (14.97 g).

The fractionation of the R7B and R20 extracts was performed using a VLC system with flash silica (230–400 mesh) as the stationary phase. Elution was conducted with 400 mL gradients of analytical‐grade organic solvents, progressively increasing the polarity of the mobile phase. The elution sequence comprised 95:5 hexane:ethyl acetate, 66:33 hexane:ethyl acetate, 33:66 hexane:ethyl acetate, 100% ethyl acetate, 100% acetone, and finally 100% methanol. This process yielded six fractions, which were subsequently evaluated for biological activity in a bioassay‐guided approach to identify the most promising fractions for further purification.

Fraction 3 from the R7B extract was selected for compound isolation using high‐performance liquid chromatography with diode array detection (HPLC‐DAD). The separation method was initially optimized on an Agilent Zorbax Phenyl analytical column (5 µm, 4.6 × 250 mm^2^) with a flow rate of 1.0 mL/min. For semi‐preparative purification, a Shimadzu LC‐6AD pump and a Phenomenex Luna RP‐C18 column (5 µm, 10 × 250 mm^2^) were utilized, operating at a flow rate of 4.0 mL/min and an injection volume of 450 µL. The elution system employed a chromatographic‐grade methanol and ultrapure water gradient (30%–100%) over 20 min. UV detection was performed at 309 nm, resulting in the resolution of seven distinct peaks. The chromatogram is presented in Figure S1A. The major peak (VI, 13.9 min) underwent further chromatographic purification, leading to the isolation of compound **1** (33.3 mg).

Fraction 4 from the R7B extract was subfractionated using a size‐exclusion chromatography process, resulting in eight subfractions (f4R7Ba to f4R7Bh). For this step, Sephadex LH‐20 (Amersham Pharmacia Biotech AB) was used as the stationary phase in a glass column with dimensions of 2.5 cm in diameter and 60 cm in height. The Sephadex was suspended in methanol (analytical grade) and packed to occupy a column height of 53 cm. The extract was dissolved in methanol and applied to the top of the column. Methanol (analytical grade) was also used as the mobile phase to elute the compounds. The separation process was monitored using thin‐layer chromatography (TLC). Visualization was performed under UV light at wavelengths of 254 and 365 nm, followed by staining with vanillin–sulfuric acid reagent. TLC analysis revealed the presence of the major compound in subfraction f4R7Bd. This subfraction was subjected to isolation by HPLC‐DAD with a semi‐preparative RP‐C18 column, using a methanol/water solvent gradient (40%–100%) at a flow rate of 4 mL/min over 20 min and an injection volume of 450 µL, yielding ten peaks (UV detection at 295 nm). The chromatogram is presented in Figure S1B. The major peak (III, 11.9 min) underwent an additional chromatographic step for purification, leading to the isolation of compound **2** (27.7 mg).

The R20 extract was fractionated by VLC under the same conditions as the R7B extract. Fraction 3 (f3R20) was selected and subfractionated using flash silica (230–400 mesh), yielding 10 subfractions (f3R20a to f3R20j). This step employed a glass column measuring 45 cm in height and 2.5 cm in diameter. The silica was suspended in methanol and packed to a height of 25 cm. The sample was solubilized in silica and applied to the prepacked column. The mobile phase initially used was 6:4 hexane:ethyl acetate, with a gradual increase in polarity up to 100% methanol. The separation process was monitored using TLC. Visualization was conducted under UV light (254 and 365 nm) and revealed with vanillin–sulfuric acid. TLC analysis revealed that the major compound was present in subfraction f3R20e. The subfraction was subjected to HPLC‐DAD using a semi‐preparative RP‐C18 column to isolate this compound further. Elution was performed with a methanol/water solvent gradient (70%–100%) at a flow rate of 4 mL/min for 20 min and an injection volume of 450 µL, collecting seven peaks (UV detection at 378 nm). The chromatogram of this process is shown in Figure S1C. The major peak (II, 9.4 min) was then further purified through an additional chromatographic step, leading to the isolation of compound **3** (94.8 mg). A schematic representation of the extraction and compound isolation process has been added in Figure S10.

### 4.5 Structural Analysis

The high‐resolution mass spectrum was acquired using a micrOTOF II mass spectrometer (Bruker Daltonics, Massachusetts, USA) featuring an electrospray ionization (ESI) source and a time‐of‐flight (TOF) analyzer. Mass spectrometer calibration was performed in positive ionization mode using sodium trifluoroacetate as the reference standard, applying enhanced quadratic calibration. An infusion pump (KD Scientific) delivered the sample at a flow rate of 0.20 mL/h. The analysis was conducted in positive ionization mode, with a drying gas flow rate of 7 L/min, a pressure of 2 bar, and a temperature of 180°C. The capillary and end‐plate voltages in the ESI source were set to 3.0 and 0.45 kV, respectively. NMR spectra were recorded at 400 MHz (9.4 T) (DRX‐400, Bruker Advance) and 500 MHz (11.7 T) (DRX‐500, Bruker), using methanol‐d4, CDCl3, and DMSO‐d6 as solvents. DMSO‐d6 and methanol‐d4 were purchased from Sigma‐Aldrich (USA). Deuterated chloroform was purchased from Sigma‐Aldrich (India).

### 4.6 Antifungal Activity Assay

The antifungal activity of the isolated compounds was evaluated using the agar dilution method [69]. The isolated compounds were solubilized in 70% ethanol and incorporated into molten PDA medium at final concentrations of 100, 50, 25, 12.5, 6.25, 3.12, and 1.56 µg/mL. The prepared medium was poured into sterile petri dishes, and mycelial discs (0.5 cm in diameter) were aseptically placed at the center of each PDA plate and incubated at 28°C for 7 days. A control group was prepared using PDA medium without the addition of the test compounds. The experiments were performed in triplicate across two independent biological replicates, resulting in a total of six replicates (*n* = 6). Fungal growth inhibition was determined by measuring the mycelial diameters in both the control and treated groups, followed by calculating the percentage of inhibition relative to the control.

### 4.7 Protease‐Inhibitory Activity Assay

Papain was used as a cysteine protease model for the protease inhibition assay. Enzymatic activity was determined by continuously monitoring the increase in fluorescence emitted by the MCA (7‐amino‐4‐methylcoumarin) residue released during the hydrolysis of the ZFR‐MCA substrate over time [70]. Kinetic enzymatic activity measurements were performed using a spectrofluorometer with excitation and emission wavelengths set at *λ*ex = 380 nm and *λ*em = 460 nm, respectively, using black 96‐well plates. To conduct the assay, 5 µL of papain (80 nM), 2 µL of dithiothreitol (500 mM), and 158 µL of 100 mM sodium acetate buffer with 5 mM EDTA (pH 5.5) were added to each well. The plate was incubated at 27°C for 5 min. Subsequently, 5 µL of either the negative control (DMSO) or the sample dissolved in DMSO at concentrations of 6, 4, and 2 mM was added to determine the concentration required to achieve approximately 100% enzymatic inhibition. This concentration was then used as the starting point for a dilution series to determine the concentration required to inhibit enzymatic activity by 50% (IC_50_). The plate was incubated again at 27°C for 5 min. Finally, 30 µL of the fluorogenic substrate ZFR‐MCA (0.6 mM) was added, and fluorescence readings were taken using the SpectraMax M3 microplate reader (Molecular Devices) for 300 s. Considering the final volume in each well was 200 µL, the effective concentrations of the samples ranged from 150 to 1.56 µM in the IC_50_ assay. To accurately determine the molar concentration of papain, the enzyme was titrated with the irreversible enzyme inhibitor E‐64 (positive control) at concentrations of 1 µM, 500 nM, 250 nM, 125 nM, 62.5 nM, and 31.25 nM. Experiments were performed in triplicate on two independent occasions, yielding a total of six replicates (*n* = 6).

### 4.8 Anti‐*Trypanosoma Cruzi* Assay

Compounds demonstrating promising enzymatic inhibition of papain were further evaluated for their activity against *T. cruzi* [71]. The assay was performed using the *T. cruzi* Tulahuen lacZ strain, which has been genetically modified to incorporate the lacZ gene responsible for encoding the enzyme β‐galactosidase. Active parasites within host cells express β‐galactosidase, an enzyme capable of cleaving the CPRG substrate (chlorophenol red‐β‐d‐galactopyranoside) to produce a red product (chlorophenol red), which is quantified by spectrophotometry. The intensity of the red color reflects the number of viable intracellular parasites at the time of measurement.

Axenic cultures of *T. cruzi* epimastigotes (strain MHOM/CH/00/Tulahuen C2, lacZ) were maintained at 28°C in LIT medium supplemented with 10% fetal calf serum (FCS). Epimastigotes in the exponential growth phase were harvested by centrifugation at 250 × *g* for 10 min, resuspended in 1 mL of Grace's insect medium supplemented with 10% FCS, and induced to differentiate into metacyclic trypomastigotes for 5–14 days. Following differentiation, the parasites were harvested and used to infect human foreskin fibroblasts (HFF‐1). HFF‐1 cells were cultured in DMEM supplemented with 10% FCS under 5% CO_2_ at 37°C. For the infection assay, HFF‐1 cells were seeded in 96‐well plates at a density of 5 × 10^4^ cells per well in 80 µL of phenol red‐free DMEM and incubated overnight. On the following day, 20 µL of a suspension of metacyclic trypomastigotes (5 × 10^5^ cells per well) was added to the wells. After 24 h of incubation, the medium was removed to eliminate noninfective parasites, and 100 µL of fresh medium was added to each well. Compounds were then added at various concentrations to determine their IC_50_ values.

The plates were incubated for 5 days at 37°C under 5% CO_2_. Benznidazole was used as a positive control. At the end of the incubation period, 50 µL of CPRG (1 mM) and the surfactant IGEPAL CA‐630 (final concentration 0.1%) was added to each well. β‐Galactosidase activity, indicative of parasite viability, caused a color change in the medium from yellow to red, which was measured at 570 nm using a microplate reader. The percentage inhibition of parasite growth was calculated relative to untreated controls.

## Author Contributions

**Gabriela de Oliveira Almeida**: conceptualization, methodology, investigation, writing – original draft preparation. **Paulo Cezar Vieira**: conceptualization, writing – review and editing, supervision, funding acquisition. **Vitor de Souza Mazucato**: methodology. **Marcia Regina von Zeska Kress**: methodology. **Adriano Defini Andricopulo**: methodology. **Gisele Barbosa**: methodology. **Renata Krogh**: methodology. **Leonardo Luiz Gomes Ferreira**: methodology. **Ludmilla Tonani**: methodology. All authors have read and agreed to the published version of the manuscript.

## Conflicts of Interest

The authors declare no conflicts of interest.

## Supporting information




**Supporting File 1**: cbdv70252‐sup‐0001‐SuppMat.pdf

## Data Availability

The data that support the findings of this study are available from the corresponding author upon reasonable request.
