# Supplementary material for: Exploring Bioactive Metabolites From Fusarium falciforme and Aspergillus terreus Isolated From Protease‐Rich Fruits: Antifungal, Antitrypanosomal, and Enzymatic Inhibitory Activities
Source: Chem Biodivers. 2025 Jul 20;22(11):e00673. doi: 10.1002/cbdv.202500673 (PMC12629174; doi:10.1002/cbdv.202500673)
Supplement: Supplementary file 1 — Supporting File 1: cbdv70252‐sup‐0001‐SuppMat.pdf [file CBDV-22-e00673-s001.pdf]

Supplementary Material

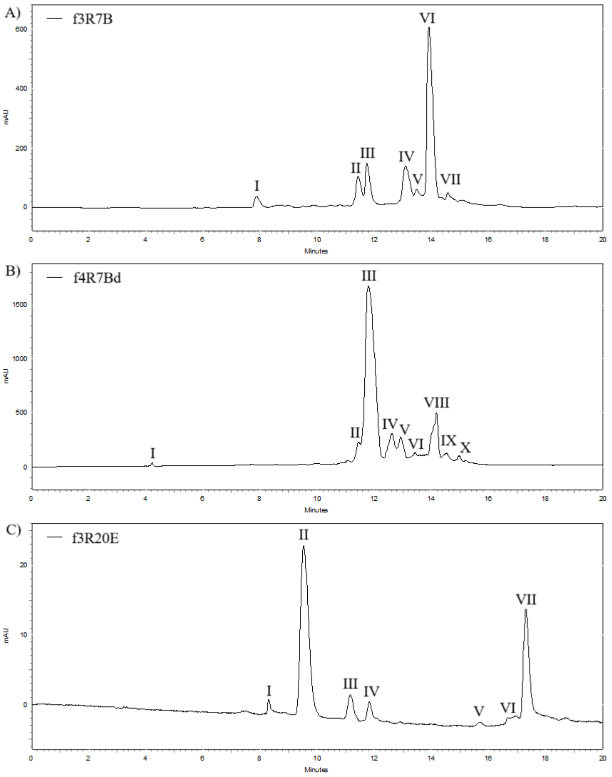

Fig. S1. High-performance liquid chromatography (HPLC) chromatograms of fractions derived from ethyl acetate (EtOAc) extracts. (A) Fraction 3 from *Fusarium falciforme* cultured on rice, detected at UV 309 nm; (B) Subfraction 4d from *F. falciforme* cultured on rice, detected at UV 295 nm; (C) Subfraction 3e from *Aspergillus terreus* cultured on rice, detected at UV 378 nm.

Code	Molecular Identification	Gene/ Region	Score	Per. Ident (%)	E value	Reference code NCBI	Code NCBI
LMC23007.2	*Fusarium falciforme*	ITS1; 5,8S; ITS2	976	100	0	KX064979.1	PV089057
		EF-1	789	99.31	0	CP155522.1	PV101038
		RPB2	1688	100	0	KF255514.1	PV101039
LMC23020	*Aspergillus terreus*	ITS1; 5,8S; ITS2	789	100	0	MT505692.1	PV089058
		caM	1011	99.28	0	EU147527.1	PV101041

β-tubulin	929	97.61	0	CP155522.1	PV1010 40

Table S1. Molecular identification of samples. The table presents molecular identification results, including sample code, gene/region, score, percentage identity, E-value, and NCBI reference codes.

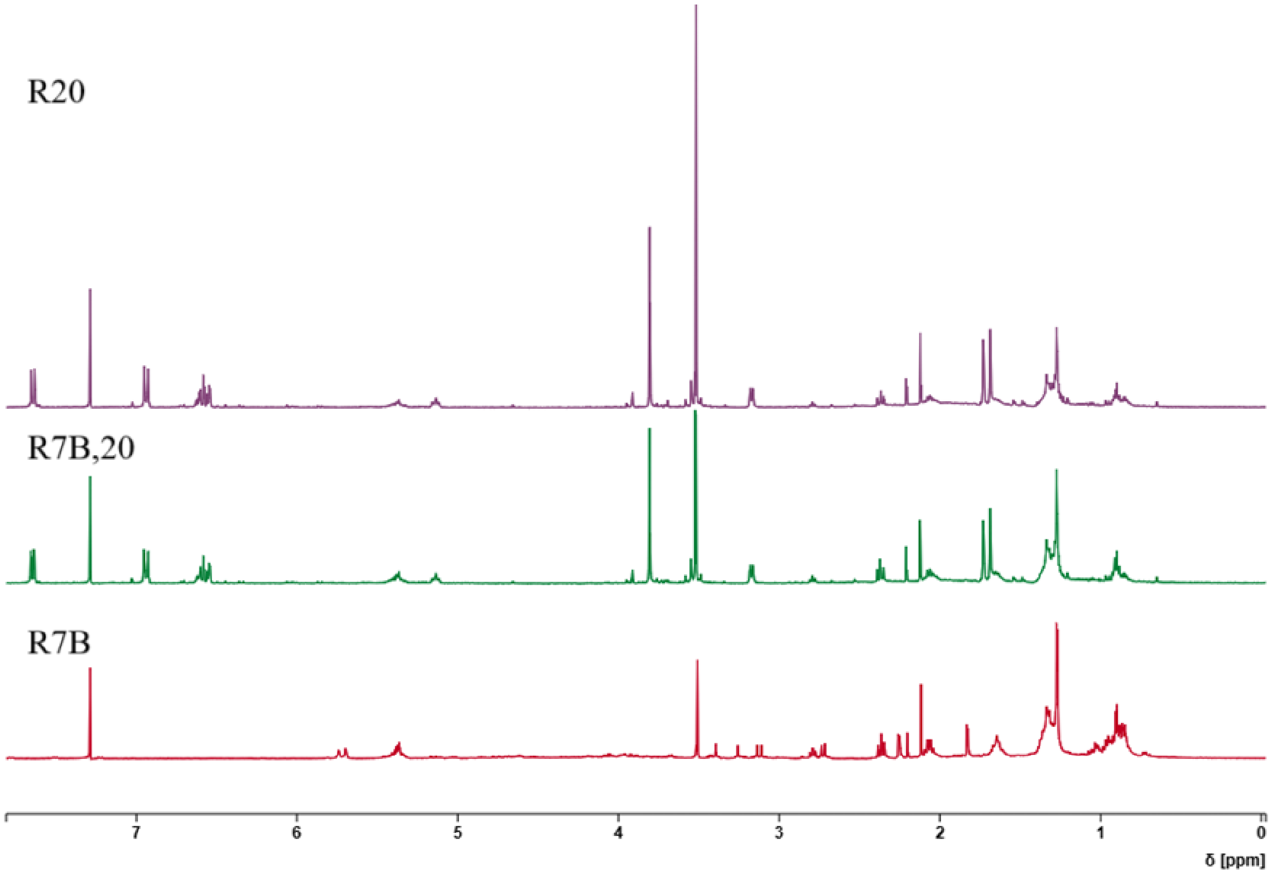

Fig. S2. ^1H NMR spectra (400 MHz) of the extracts R20 (purple), R7B,20 (green), and R7B (red) after triglyceride removal from the rice medium. The spectra were recorded in $CDCl_3$ as the solvent and processed using the NMRium software.

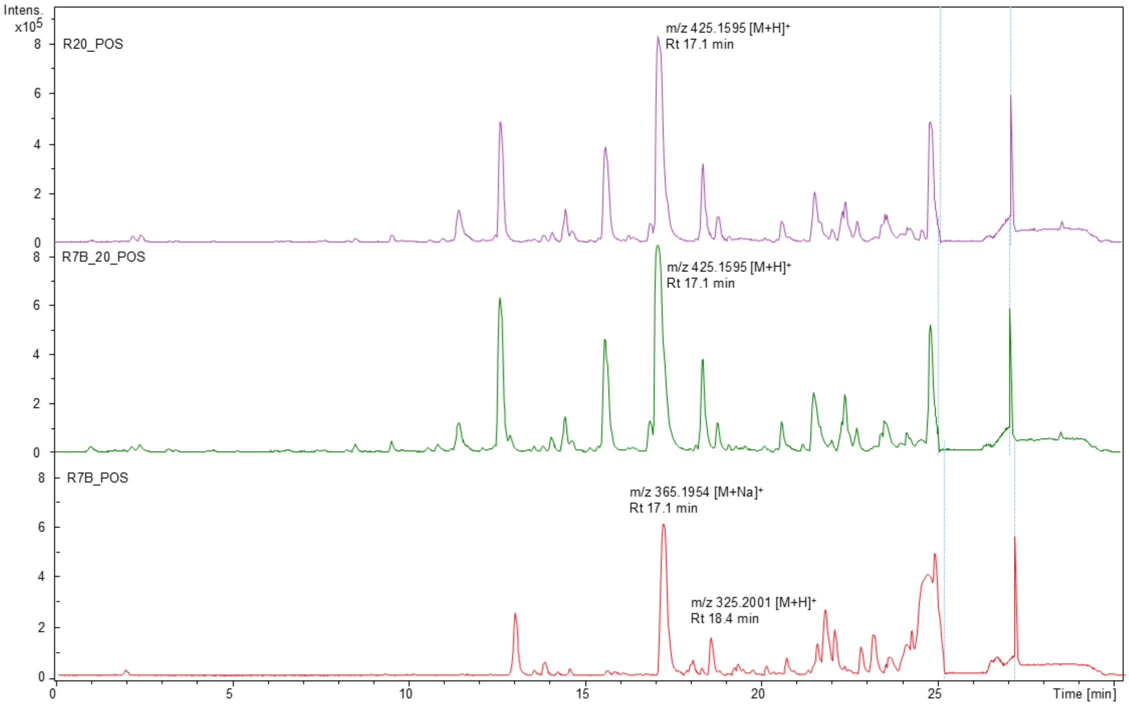

Fig. S3. High-resolution LC-MS base peak chromatograms of the extracts R20 (purple), R7B,20 (green), and R7B (red) after triglyceride removal from the rice medium. The analyses were performed in positive ionization mode using a C18 reversed-phase column. The chromatograms were processed and visualized using DataAnalysis software.

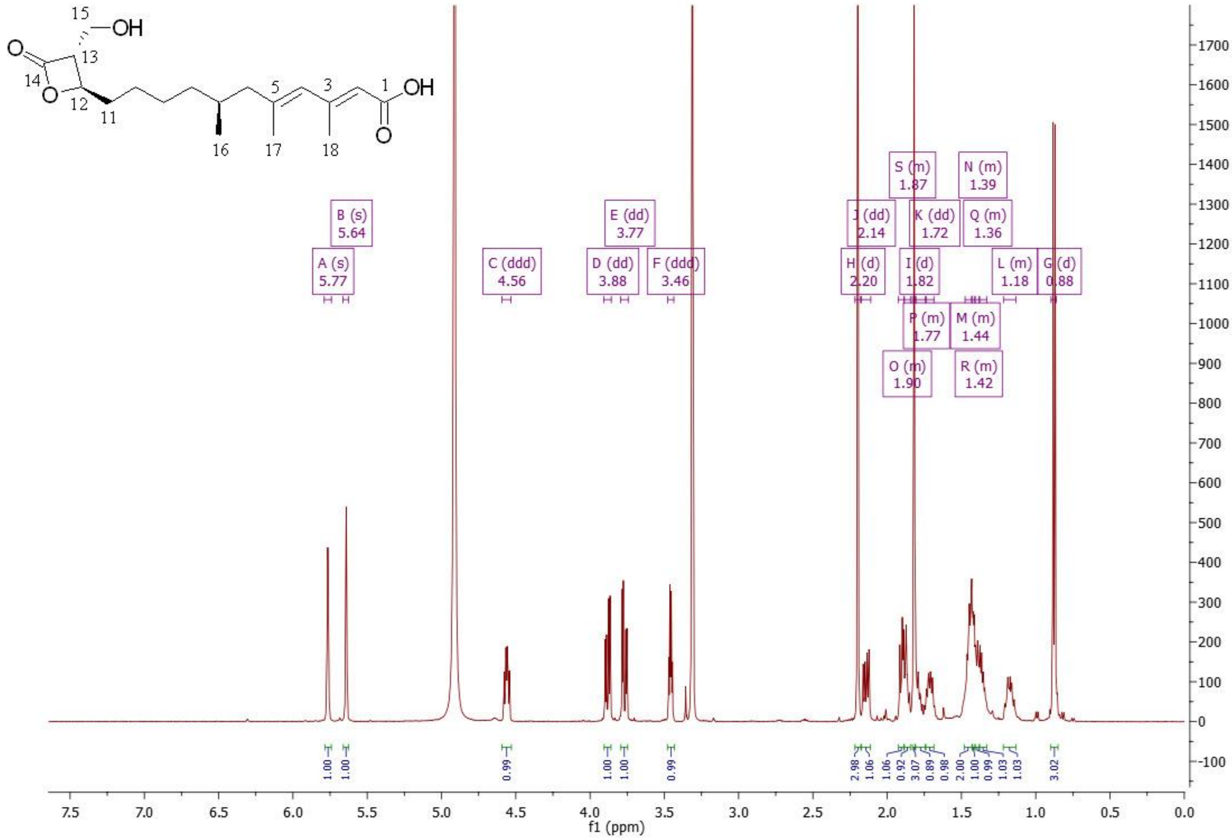

Fig. S4. ¹H NMR spectrum of **compound 1**, isolated from fraction 3 of the ethyl acetate extract of the axenic *F. solani* culture in rice. The spectrum was recorded in methanol-d_4 at 500 MHz and processed using the MestReNova software. The upper

left corner shows the structure of hymeglusin, with the carbon numbering corresponding to the chemical shifts presented in the table 1.

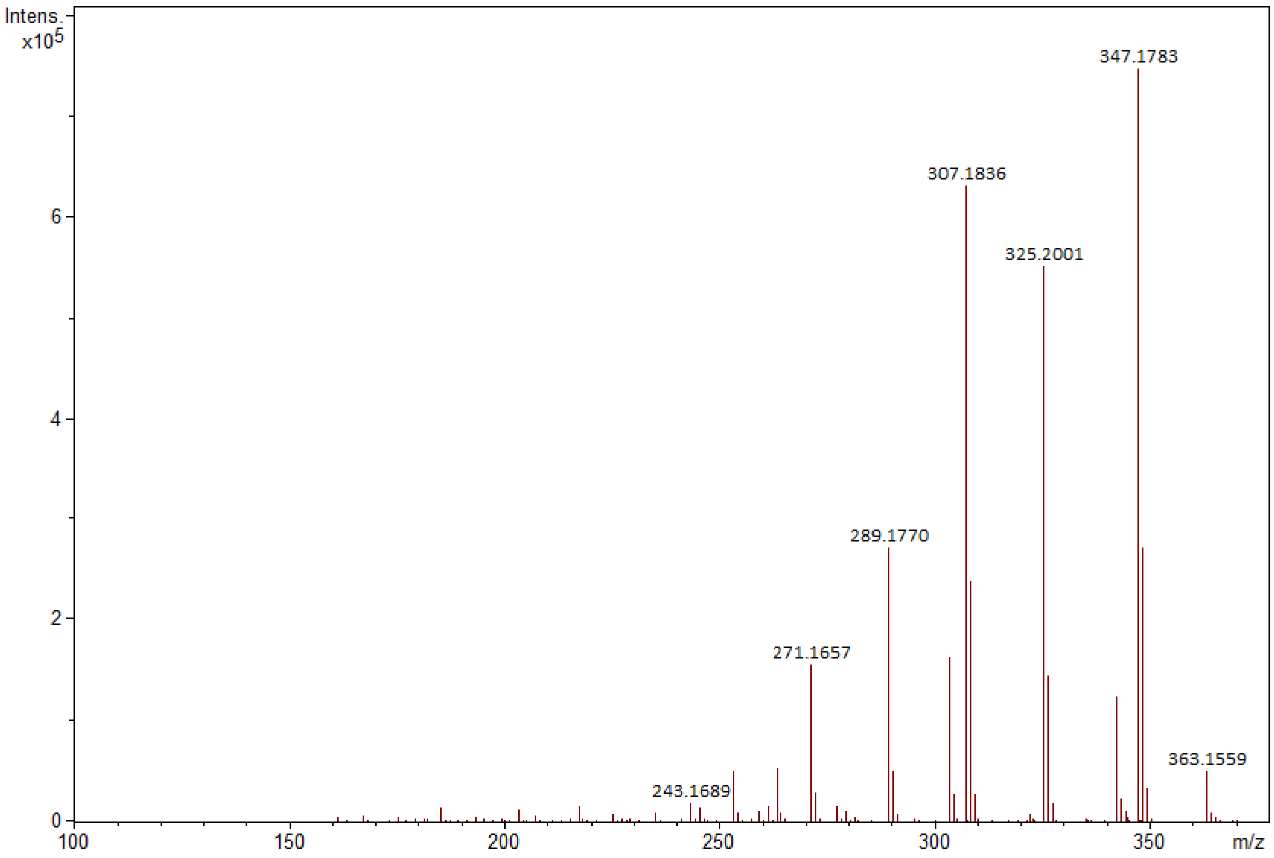

Fig. S5. HRESIMS (+) of hymeglusin (**1**).

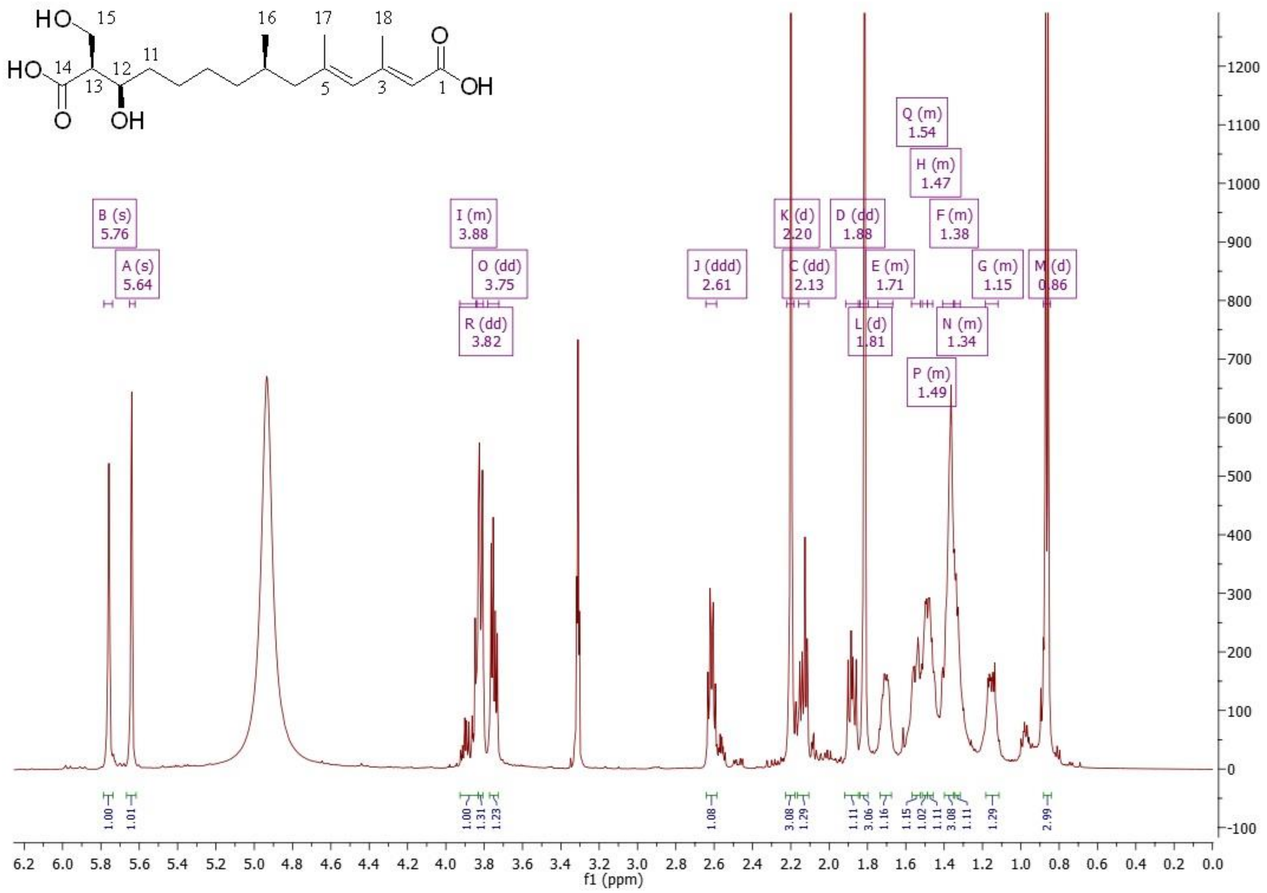

Fig. S6. ^{1}H NMR spectrum of **compound 2**, isolated from fraction 4 of the ethyl acetate extract of the axenic *F. solani* culture in rice. The spectrum was recorded in methanol-d_4 at 500 MHz and processed using the MestReNova software. The upper left corner shows the structure of fusaridioic acid A, with the carbon numbering corresponding to the chemical shifts presented in the table 1.

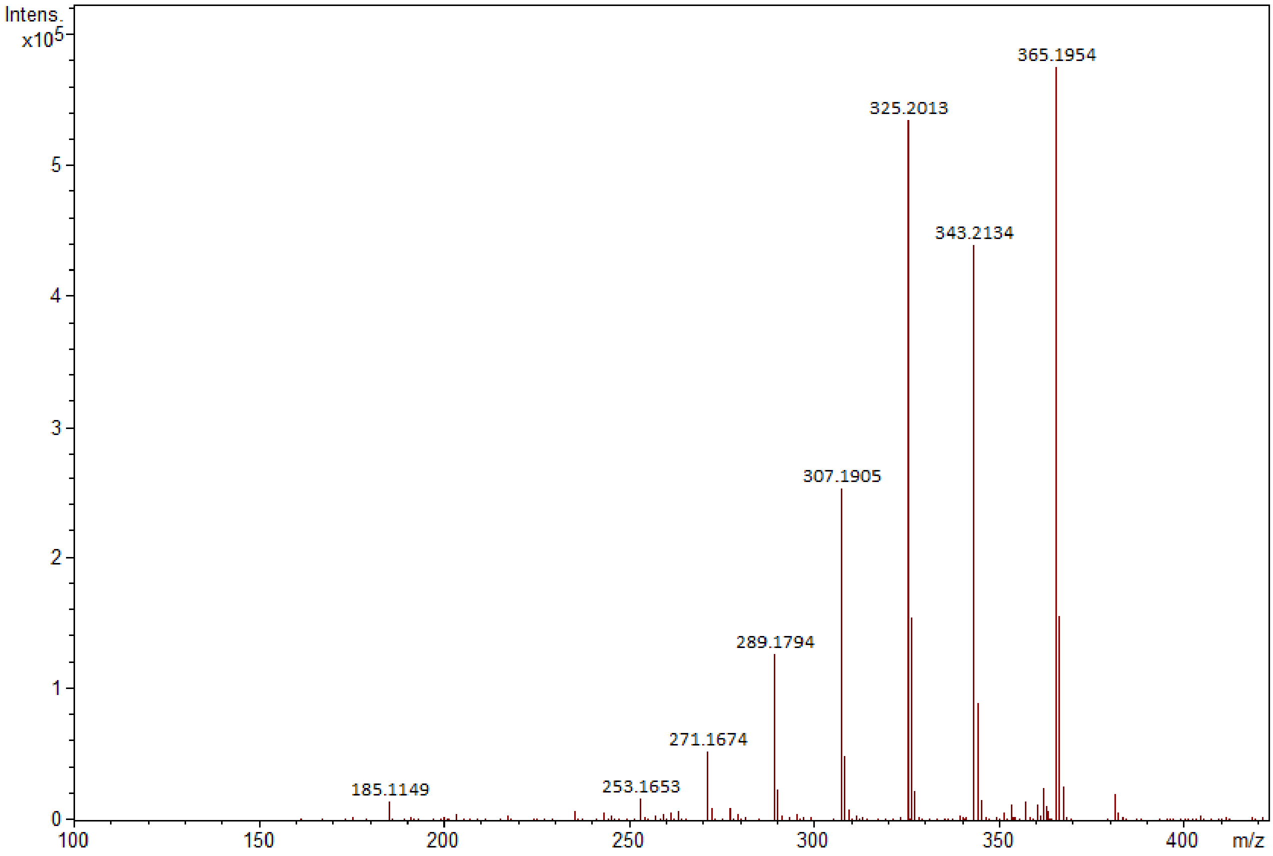

Fig. S7. HRESIMS (+) of fusaridioic acid A (**2**).

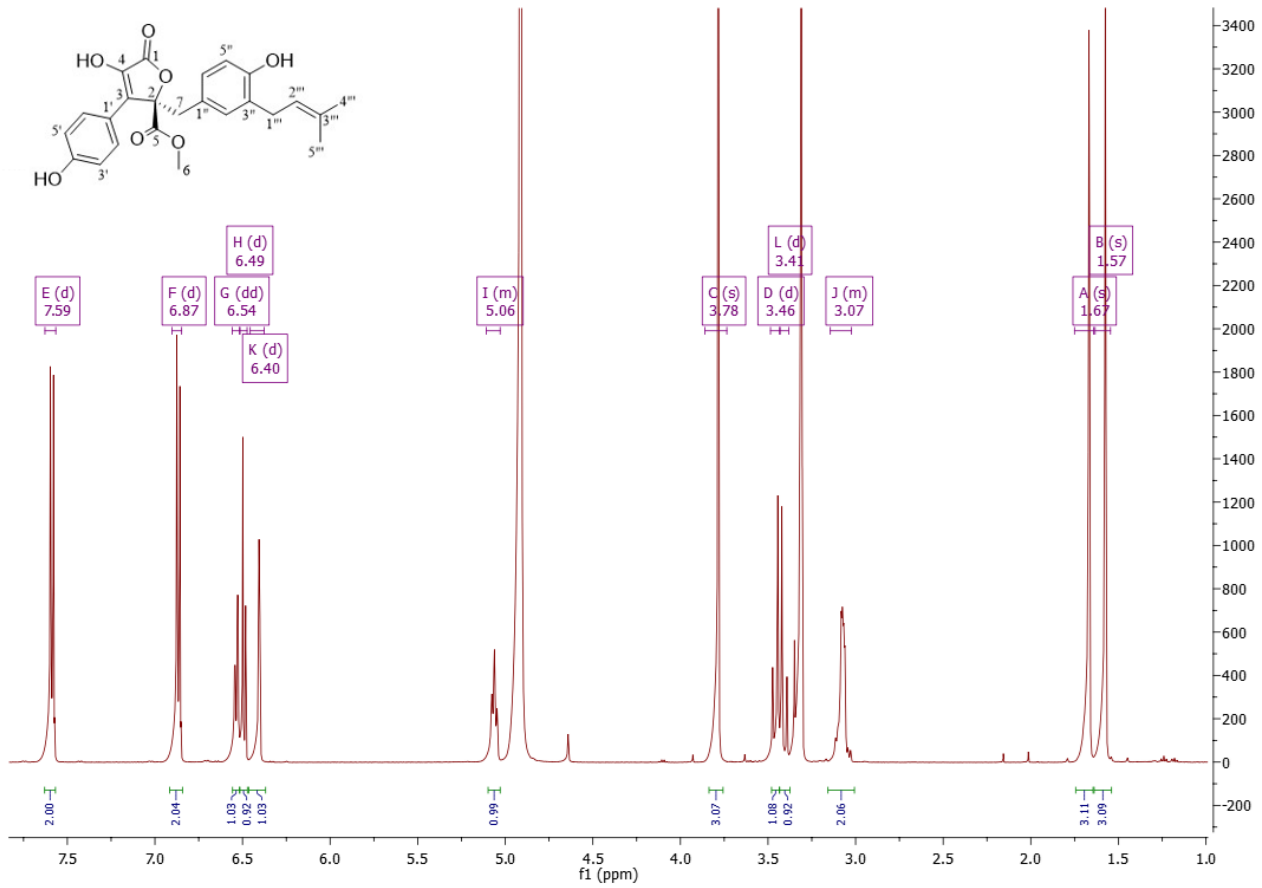

Fig. S8. ^{1}H NMR spectrum of **compound 3**, isolated from fraction 3 of the ethyl acetate extract of the axenic *A. terreus* culture in rice. The spectrum was recorded in methanol-d$_4$ at 500 MHz and processed using the MestReNova software. The upper left corner shows the structure of butyrolactone I, with the carbon numbering corresponding to the chemical shifts presented in the table 1.

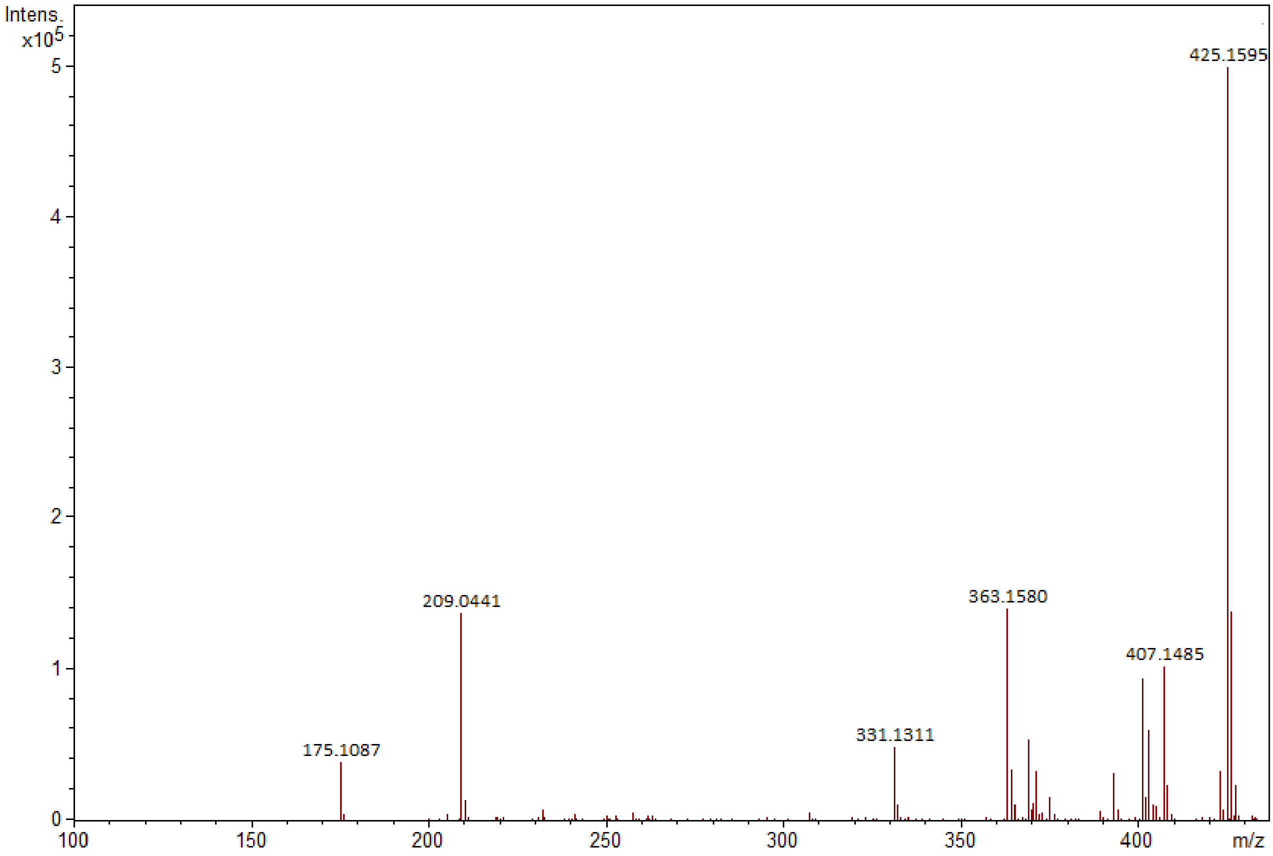

Fig. S9. HRESIMS (+) of butyrolactone I (**3**).

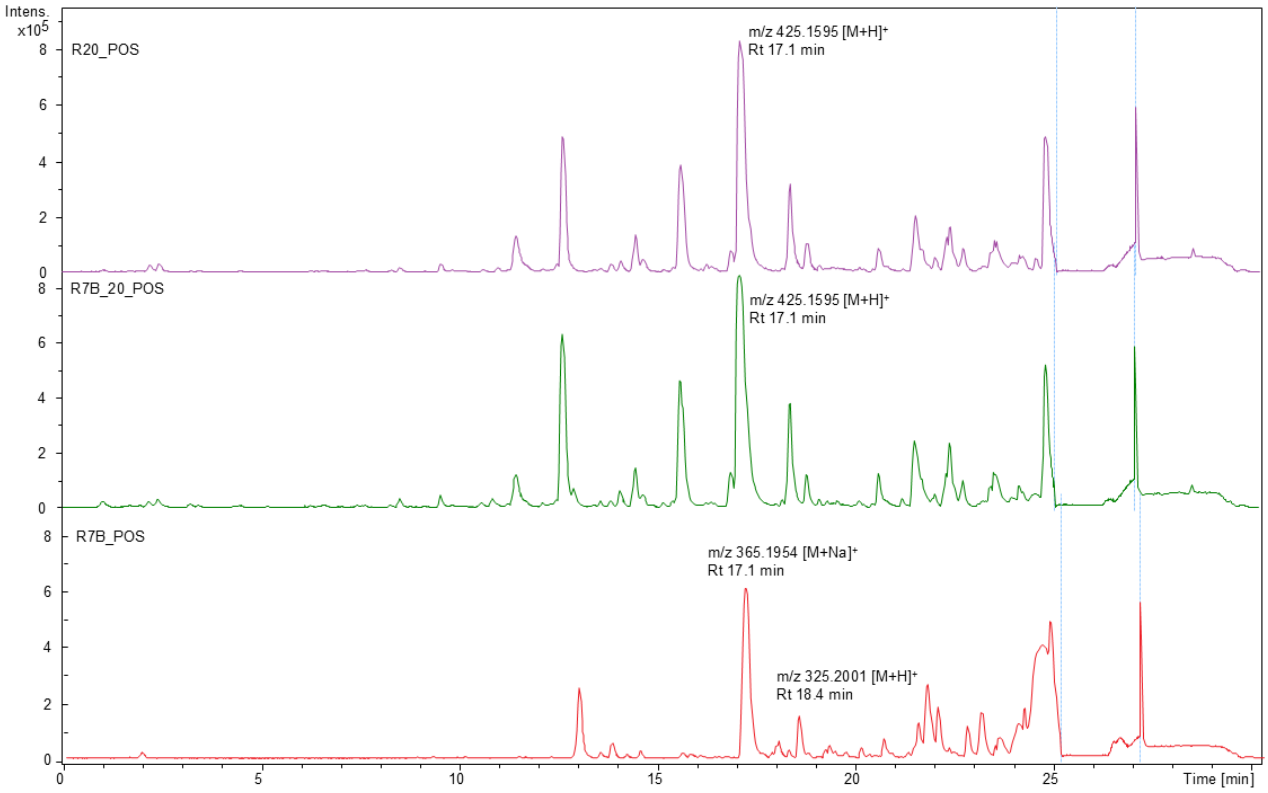

Fig. S10. Schematic representation of the extraction, fractionation, and isolation processes employed in this study. The diagram summarizes the main steps from crude extract preparation to the isolation of pure compounds **1-3**.

Atom	Compound 1 (Hymeglusin)	Compound 2 (Fusaridioic Acid A)	Compound 3 (Butyrolactone I)
**2**	5.64 (br.s, 1H)	5.64 (br.s, 1H)	
**4**	5.77 (br.s, 1H)	5.76 (br.s, 1H)	
**6**			3.78 (s, 3H)
**6a**	2.14 (dd, J =13.1, 6.5 Hz, 1H)	2.13 (dd, J = 13.3, 5.9 Hz, 1H)	
**6b**	1.72 (dd, J =13.1, 6.5 Hz, 1H)	1.88 (dd, J = 13.3, 8.4 Hz, 1H)	
**7**	1.77 (m, 1H)	1.71 (m, 1H)	
**7a**			3.41 (d, J = 14.7 Hz, 1H)
**7b**			3.46 (q, J = 14.7 Hz, 1H)
**8a**	1.18 (m, 1H)	1.15 (m, 1H)	
**8b**	1.42 (m, 1H)	1.34 (m, 1H)	
**9a**	1.36 (m, 1H)		
**9b**	1.39 (m, 1H)		
**10**	1.44 (m, 2H)		
**9a, 9b, 10a**		1.38 (m, 3H)	
**10b**		1.49 (m, 1H)	
**11a**	1.90 (m, 1H)	1.47 (m, 1H)	
**11b**	1.87 (m, 1H)	1.54 (m, 1H)	
**12**	4.56 (ddd, J = 7.4, 6.1, 4.1 Hz, 1H)	3.88 (m, 1H)	

**13**	3.46 (ddd, J = 4.7, 4.1, 3.6 Hz, 1H)	2.61 (ddd, J = 11.3, 8.0, 5.4 Hz, 1H)	
**15a**	3.88 (dd, J = 11.9, 4.7 Hz, 1H)	3.82 (dd, J = 10.6, 8.0 Hz, 1H)	
**15b**	3.77 (dd, J = 11.9, 3.6 Hz, 1H)	3.75 (dd, J = 10.6, 5.4 Hz, 1H)	
**16**	0.88 (d, J = 6.6 Hz, 3H)	0.86 (d, J = 6.6 Hz, 3H)	
**17**	1.82, d (1.1 Hz, 3H)	1.81 (d, J = 1.1 Hz, 3H)	
**18**	2.20, d (1.1 Hz, 3H)	2.20 (d, J = 1.1 Hz, 3H)	
**2'/6'**			7.59 (d, J = 8.9 Hz, 2H)
**3'/5'**			6.87 (d, J = 8.9 Hz, 2H)
**2"**			6.40 (d, J = 2.0 Hz, 1H)
**5"**			6.49 (d, J = 8.2 Hz, 1H)
**6"**			6.54 (dd, J = 8.2, 2.0 Hz, 1H)
**1'"**			3.07 (m, 2H)
**2'"**			5.06 (m, 1H)
**4'"**			1.67 (s, 3H)
**5'"**			1.57 (s, 3H)

Table S2. Summary of ^{1}H NMR (600 MHz, metanol-d_4) spectroscopic data for compounds **1**-**3**.
